# Comparing the postoperative refractive predictability of Pentacam HR and IOLMaster 500 after a multifocal intraocular lens implantation

**DOI:** 10.5935/0004-2749.20200026

**Published:** 2020

**Authors:** Newton Andrade Junior, Wilson Takashi Hida, André Marcio Vieira Messias, João Marcelo Lyra, Carlos André Mont’Alverne Silva, Milton Ruiz Alves

**Affiliations:** 1 Hospital de Olhos Leiria de Abdrade, Fortaleza, CE, Brazil; 2 Hospital Oftalmológico de Brasília, Brasília, DF, Brazil; 3 Departamento de Oftalmologia, Otorrinolaringologia e Cirurgia de Cabeça e Pescoço, Faculdade de Medicina, Universidade de São Paulo, Ribeirão Preto, SP, Brazil; 4 Hospital VER, Maceió, AL, Brazil; 5 Departamento de Oftalmologia, Faculdade de Medicina, Universidade de São Paulo, São Paulo, SP, Brazil

**Keywords:** Biometry, Cataract, Interferometry, Lenses, intraocular, Multifocal intraocular lenses, Biometria, Catarata, Interferometria, Lentes intraoculares, Lentes intraoculares multifocais

## Abstract

**Purpose:**

To compare the postoperative refractive predictability of IOLMaster 500 and Pentacam HR on the basis of keratometry and anterior chamber depth values in eyes with an indication for multifocal intraocular lens (IOL) implantation.

**Methods:**

This was a retrospective study conducted on 118 eyes treated with phacoemulsification and multifocal intraocular lens implantation. Only the eyes that achieved emmetropia in the dynamic refraction performed on postoperative day 30 were included. Haigis’ formula was used in each case to calculate the intraocular lens power, and the intraocular lens with the target refraction closest to emmetropia was implanted. Four lens calculation scenarios were tested by combining keratometry and anterior chamber depth measurements obtained using the two devices.

**Results:**

IOLMaster 500 and Pentacam HR differed with regard to mean keratometry (∆ 0.07 ± 0.03 D; p=0.0065) and anterior chamber depth (∆ 0.08 ± 0.01 mm; p<0.001). In the analysis of covariance, the following differences were obtained using the Haigis’ formula when confronted with the biometric values obtained by inserting keratometry and anterior chamber depth values, respectively: Penta/IOL x IOL/Penta (0.13 ± 0.03; p<0.0001); Penta/Penta × IOL/Penta (0.13 ± 0.03; p<0.0001); Penta/IOL × IOL/IOL (0.11 ± 0.03; p=0.001); Penta/ Penta × IOL/IOL (0.11 ± 0.03; p=0.002); IOL/IOL × IOL/Penta (0.02 ± 0.03; p=0.865); and Penta/IOL × Penta/Penta (0.002 ± 0.03; p=0.99). The difference was smaller when measuring the anterior chamber depth using the IOLMaster 500, regardless of which device was used to measure keratometry.

**Conclusions:**

Pentacam HR significantly differed from IOLMaster 500 when calculating keratometry. As regards the anterior chamber depth, the two devices were equally accurate.

## Abstract (pt)

**Objetivo:**

Comparar a previsibilidade refrativa pós-operatória do IOLMaster 500 e Pentacam HR com base nos valores de ceratometria e profundidade de câmara anterior nos olhos com indicação de implante de lentes intraoculares multifocais.

**Métodos:**

Estudo retrospectivo realizado em 118 olhos tratados com facoemulsificação e implante de lentes intraoculares multifocal. Apenas os olhos que atingiram a emetropia na refração dinâmica no 30º dia pós-operatório foram incluídos. A fórmula de Haigis foi usada em cada caso para calcular o poder das lentes intraoculares, e a lente intraocular com a refração alvo mais próxima da emetropia foi implantada. Cenários de cálculo de quatro lentes foram testados pela combinação de medidas de ceratometria e profundidade de câmara anterior obtidas usando os dois dispositivos.

**Resultados:**

IOLMaster 500 e Pentacam HR diferiram quanto à média de ceratometria (∆ 0,07 ± 0,03 D; p=0,0065) e profundidade de câmara anterior (∆ 0,08 ± 0,01 mm; p<0,001). Na análise da covariância, as seguintes diferenças foram obtidas usando a fórmula de Haigis quando confrontadas com os valores biométricos obtidos pela inserção dos valores de ceratometria e profundidade de câmara anterior, respectivamente: Penta/IOL x IOL/Penta (0,13 ± 0,03; p<0,0001); Penta/Penta x IOL/Penta (0,13 ± 0,03; p<0,0001); Penta/IOL x IOL/IOL (0,11 ± 0,03; p=0,001); Penta/Penta x IOL/IOL (0,11 ± 0,03; p=0,002); IOL/IOL x IOL/Penta (0,02 ± 0,03; p=0,865); Penta/IOL x Penta/Penta (0,002 ± 0,03; p=0,99). A diferença foi menor ao medir a profundidade da câmara anterior usando o IOLMaster 500, independentemente de qual dispositivo foi usado para medir a ceratometria.

**Conclusões:**

O Pentacam HR diferiu significativamente do IOLMaster 500 no cálculo de ceratometria. Quanto à profundidade da câmara anterior, os dois dispositivos foram igualmente precisos.

## INTRODUCTION

Over the past 30 years, several formulas and devices have been proposed to improve the refractive predictability and reduce refractive errors after a cataract sur gery^(1-3)^. As calculations are based on preoperative eye dimensions, such as axial length (AL), keratometry (K), and anterior chamber depth (ACD), careful eye mea surements should be performed for accuracy. The refractive outcome is predicted based on three main factors: i) uneventful surgery with a well-centered in-the-bag implanted intraocular lens (IOL); ii) accuracy of preoperative biometric data (AL, ACD, and K); and iii) predictability of the formula used to calculate IOL power, using optimized IOL constants^(4-13)^. For example, a 1-mm deviation in the corneal diameter, axial diameter, or ACD has been reported to result in a postoperative refractive error of 5.7 D, 2.7 D, or 1.5 D, respectively^(11)^.

Postoperative refraction predictability is even more important when implanting multifocal IOLs. IOLMaster 500 (Carl Zeiss Meditec AG, Jena, Germany) is the gold standard for biometric measurements and calculations; however, some studies have questioned the accuracy of its generated K measurements (using data from six light reflections at a 2.3-mm diameter), especially when compared to Pentacam HR (Oculus Optikgeräte GmbH, Wetzlar, Germany), which uses a Scheimpflug camera (180°) and a monochromatic slit-light source combined with a static camera^(14)^. Reitblat et al.^(15)^ recently compared the accuracy of IOLMaster and Lenstar in patients undergoing multifocal IOL implantation (SN6AD1; Alcon Laboratories, Inc., Fort Worth, TX, USA) and concluded that both devices were highly accurate, when using similar measurement methods.

Therefore, this study aimed to compare the postoperative refractive predictability of IOLMaster 500 and Pentacam HR based on K and ACD values in the eyes implanted with multifocal IOLs.

## METHODS

The study was conducted at the Cataract Division of Hospital Oftalmológico de Brasilia, Brazil, with the study protocol complying the tenets of the Declaration of Helsinki and approved by the institutional ethics committee.

### Patients and contraindications for multifocal IOL

Medical records of all eyes submitted for cataract surgery with multifocal IOL implantation (AcrySof IQ ReSTOR SN6AD1, Alcon, USA) between January 2014 and October 2015 were retrospectively reviewed. Eligible participants were all patients aged 45-65 years with bilateral senile cataract, corneal astigmatism of <1.00 diopter in both eyes; pupil diameter of at least 3.5 mm under mesopic conditions; and absence of other eye diseases, topical hypotensive medication use, and previous eye surgery. Intraoperative and postoperative exclusion criteria were doubts about IOL implantation within the capsular bag or capsulorhexis described as >0.5 mm as verified by the slit-lamp examination, and patients who did not achieve emmetropia in the dynamic refraction performed on postoperative day 30. Because postoperative ACD was not measured, the study was designed to analyze only the eyes that achieved emmetropia in the dynamic refraction performed on postoperative day 30.

The main contraindications for multifocal IOL implantation in this study were:

- History of ocular surgery.
- Systemic changes capable of interfering with postoperative healing (e.g., diabetes mellitus, autoimmune conditions, connective tissue disorders).
- Preexisting ocular disease compromising visual acuity (e.g., herpetic ocular disease, moderate or severe dry eye syndrome, uveitis, glaucoma, retinal disorders).
- Incomplete records with regard to the study parameters
- Macular changes indicating imminent central vision loss (age-related macular degeneration, macular edema, macular hole, epiretinal membrane).
- Corneal changes interfering with K (pterygium, scars, other opacities).

### Surgical procedure

All surgical procedures were performed by a single experienced surgeon (WTH) at a surgical center following the standardized surgical technique. Under topical anesthesia, a clear self-sealing corneal 2.75-mm incision was made in the steep meridian, followed by continuous circular capsulorhexis and hydrodissection with 1% lidocaine without preservatives diluted in 10 mL of balanced saline solution. Then, the soft-shell technique was performed using Celoftal^®^ (hydroxypropyl methylcellulose; Alcon Laboratories, Fort Worth, TX, USA) and cohesive Provisc^®^ (sodium hyaluronate 1%; Alcon Laboratories, Fort Worth, TX, USA), whereas conventional phacoemulsification was performed using an Infiniti^®^ system (Alcon Laboratories, Fort Worth, TX, USA) with an IOL implanted in the capsular bag using a Royale^®^ injector (ASICO, Westmont, IL, USA).

Postoperatively, a fluoroquinolone (moxifloxacin 0.5%, Vigamox^®^; Alcon Laboratories, Fort Worth, TX, USA) was topically administered every 6 h for 7 days along with a topical corticosteroid (dexamethasone 1%, Maxidex^®^; Alcon Laboratories, Fort Worth, TX, USA), initially 1 drop every 4 h, and gradually tapered over 30 days.

### Measurements and calculations

The analysis included visual acuity with and without correction, biomicroscopy, specular microscopy, retinal mapping, and preoperative measurements obtained with IOLMaster 500 (Zeiss, Germany) and Pentacam HR (Oculus, Germany). As only the eyes that achieved emmetropia on postoperative day 30 were analyzed, the dynamic refraction performed on postoperative day 30 was used as a reference when comparing the postoperative refractive predictability of IOLMaster 500 and Pentacam HR based on K and ACD values. Haigis’ formula was used in each case to calculate the IOL power, and the IOL with the target refraction closest to emmetropia was implanted. Four lens calculation scenarios were tested by combining K and ACD measurements obtained using the two devices: K and ACD measured with IOLMaster 500; K and ACD measured with Pentacam HR; K measured with IOLMaster 500/ACD measured with Pentacam HR; and K measured with Pentacam HR/ACD measured with IOLMaster 500 (Table 1).

**Table 1:** Calculation of intraocular lens power using Haigis’ formula (n = 118 eyes)

Lens calculation scenario	ACD IOL	ACD penta	Best IOL	Ideal best	Ideal IOL	K IOL	K penta
K IOL × ACD IOL	3.11 ± 0.03	3.19 ± 0.03	21.73 ± 0.24	0.18 ± 0.01	21.56 ± 0.24	44.03 ± 0.12	43.95 ± 0.12
K IOL × ACD Penta	3.11 ± 0.03	3.19 ± 0.03	21.73 ± 0.24	0.15 ± 0.01	21.59 ± 0.24	44.03 ± 0.12	43.95 ± 0.12
K Penta × ACD IOL	3.11 ± 0.03	3.19 ± 0.03	21.73 ± 0.24	0.28 ± 0.03	21.66 ± 0.24	44.03 ± 0.12	43.95 ± 0.12
K Penta × ACD Penta	3.11 ± 0.03	3.19 ± 0.03	21.73 ± 0.24	0.28 ± 0.03	21.69 ± 0.24	44.03 ± 0.12	43.95 ± 0.12

K= keratometry; ACD= anterior chamber depth; Penta= Pentacam^®^ HR; IOL= IOLMaster^®^ 500. Values are presented as mean ± standard deviation.

ACD was measured from the corneal epithelium to the anterior lens capsule and from the corneal endothelium to the anterior lens capsule^(16)^. To ensure comparability between the measurements obtained with the two devices, the central corneal thickness was measured from the epithelium to the endothelium when using Pentacam HR, and this value was added to the ACD endothelium-to-lens value (“AD” on the display). This result is equivalent to the ACD epithelium-to-lens value calculated by IOLMaster 500.

### Statistical analysis

Paired *t*-test and Bland-Altman plot analysis were used to compare the K and ACD measured with the two devices. The analysis of covariance was used to determine the influence of AL and each measuring device in order to include all effects in the model, and then the Tukey’s HSD test was subsequently performed. The level of statistical significance was set at p<0.05.

## RESULTS

A total of 118 operated eyes (M=55/F=63) that achieved emmetropia in the dynamic refraction on postoperative day 30 were included in this study. The mean patient age, nuclear classification, and preoperative visual acuity were 62.3 years, 2 (N2), and 0.49 without correction and 0.89 with correction, respectively (expressed in logMAR and measured using the Early Treatment Diab etic Retinopathy Study table). No intraoperative com plications were observed.

**Figure 1:**
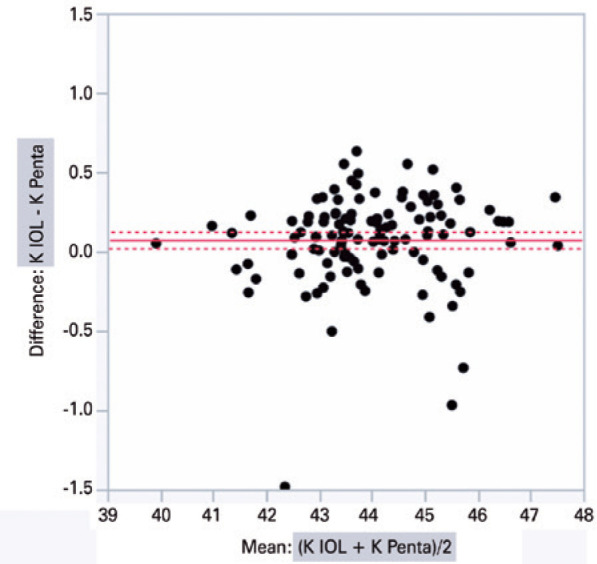
Graphical analysis of paired differences in keratometry (K) measurements obtained with IOLMaster 500 (IOL) and Pentacam HR (Penta).

**Figure 2:**
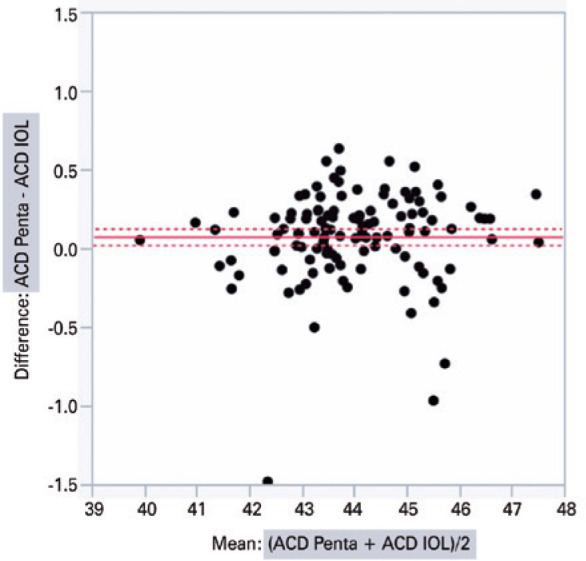
Graphical analysis of paired differences in the anterior chamber depth (ACD) measurements obtained with IOLMaster 500 (IOL) and Pentacam HR (Penta).

IOLMaster 500 and Pentacam HR significantly differed with regard to the mean K (44.03 ± 1.34 D vs. 43.95 ± 1.32 D; intra-individual difference of 0.07 ± 0.02 D; p<0.001) and ACD (3.11 ± 0.35 mm vs. 3.19 ± 0.35 mm; intra-individual difference of 0.08 ± 0.01 mm; p<0.001), respectively. The graphical analysis of paired differences in K measurements obtained with IOLMaster 500 and Pentacam HR is shown in figure 1. Likewise, the graphical analysis of paired differences in ACD measurements obtained with the two devices is shown in figure 2.

The analysis of covariance produced the following values when comparing the biometric results of different combinations of K and ACD from Pentacam HR and IOLMaster 500, respectively, inserted according to the Haigis’ formula: PENTA/IOL × IOL/PENTA (0.13 ± 0.03, p<0.0001); PENTA/PENTA × IOL/PENTA (0.13 ± 0.03, p<0.0001); PENTA/IOL × IOL/IOL (0.11 ± 0.03, p=0.001); PENTA/PENTA × IOL/IOL (0.11 ± 0.03, p=0.002); IOL/ IOL × IOL/PENTA (0.02 ± 0.03, p=0.865); and PENTA/ IOL × PENTA/PENTA (0.002 ± 0.03, p=0.99) (Table 2). The two columns in the left show the device used to measure K and ACD.

**Table 2:** Differences between mean K and ACD values from Pentacam HR and IOLMaster 500 based on analysis of covariance (n=118 eyes)

Mean K/ACD	Mean K/ ACD	Difference^*^	SE	Lower Upper CL CL p-value		
Penta/IOL	IOL/Penta	0.14	0.03	0.06	0.21	<0.0001
Penta/Penta	IOL/Penta	0.13	0.03	0.06	0.21	<0.0001
Penta/IOL	IOL/IOL	0.11	0.03	0.04	0.19	0.0012
Penta/Penta	IOL/IOL	0.11	0.03	0.04	0.19	0.0016
IOL/IOL	IOL/Penta	0.03	0.03	-0.06	0.10	0.8654
Penta/IOL	Penta/Penta	0.01	0.03	-0.08	0.08	0.9967

K= keratometry; ACD= anterior chamber depth; SE= standard error of the difference; CL= confidence limit; Penta= Pentacam^®^ HR; IOL= IOLMaster^®^ 500.

* Difference between columns 1 and 2. Note that the difference was significantly smaller when K was measured with IOLMaster, regardless of which device was used to measure ACD.

The difference was smaller when ACD was measured with IOLMaster 500, regardless of which device used to measure K.

## DISCUSSION

To improve the accuracy of biometric calculations, fourth-generation formulas, such as Haigis’ formula^(1,17)^, include not only K and AL but also ACD^(17)^. The more accurately these variables are measured, the greater the postoperative refractive predictability provided in the formula. Several measuring methods and devices are available; however, systematic differences have been observed between their results^(17,18)^. Currently, Pentacam HR significantly differed from IOLMaster 500 when calculating K. As regards ACD, the two devices were equally accurate.

Several authors have shown that coherence tomography generates higher ACD values than IOLMaster^(9,19,20)^. Previous studies also demonstrated that ACD values were significantly greater with Pentacam than with IOLMaster or Orbscan^(12,14,18,21,22)^. This is supported by our finding of a positive difference of 0.08 ± 0.01 mm in ACD when using Pentacam HR. As for K, IOLMaster and Pentacam are reported to generate similar values in the central 4.5 mm; however, the two devices differed by 0.07 ± 0.02 D in this study.

Haigis’ formula was developed to measure with IOLMaster, suggesting that this is the most appropriate technology for biometric calculations. However, after introducing the Pentacam HR technology (rotational Scheimpflug camera with controlled fixation making a detailed 3D scan of the anterior segment), anterior chamber and corneal measurements were expected to become more accurate, positively impacting the postoperative refractive predictability^(1,9,17,18,22-25)^.

This study has several limitations that should be addressed. Eighty eligible eyes submitted for cataract surgery with multifocal IOL implantation during the study period were not included in the study because they did not achieve emmetropia in the dynamic refraction on postoperative day 30. To determine the lens power in the IOL plane, postoperative ACD should have been considered. However, postoperative ACD was not measured to make this correction and achieve the accuracy required in this study. Therefore, only 118 eyes that achieved emmetropia on postoperative day 30 were included. The 118 analyzed eyes were also included based on K and ACD measurements of the IOLMaster 500, which can be considered bias in the present study. Therefore, further studies should be conducted to correlate patients who had spherical equivalent was different from 0 D in the dynamic refraction on postoperative day 30. The importance of correlating the effective measurement of postoperative lens with this residual refraction should also be emphasized in future studies.

In conclusion, within the limitations in this study, the biometric calculations obtained from K measurements with Pentacam HR and IOLMaster 500 had a disagreement. However, for ACD measurements, the two devices were equally accurate.

## References

[1] Miraftab M, Hashemi H, Fotouhi A, Khabazkhoob M, Rezvan F, Asgari S. (2014). Effect of anterior chamber depth on the choice of intraocular
lens calculation formula in patients with normal axial
length. Middle East Afr J Ophthalmol.

[2] Sahin A, Hamrah P. (2012). Clinically relevant biometry. Curr Opin Ophthalmol.

[3] Tappeiner C, Rohrer K, Frueh BE, Waelti R, Goldblum D. (2010). Clinical comparison of biometry using the non-contact optical low
coher ence reflectometer (Lenstar LS 900) and contact ultrasound biometer
(Tomey AL-3000) in cataract eyes. Br J Ophthalmol.

[4] Behndig A, Montan P, Lundström M, Zetterström C, Kugelberg M. (2014). Gender differences in biometry prediction error and intra-ocular
lens power calculation formula. Acta Ophthalmol.

[5] Rönbeck M, Lundström M, Kugelberg M. (2011). Study of possible predictors associated with self-assessed visual
function after cataract surgery. Ophthalmology.

[6] Norrby S. (2008). Sources of error in intraocular lens power
calculation. J Cataract Refract Surg.

[7] Preussner PR, Olsen T, Hoffmann P, Findl O. (2008). Intraocular lens calculation accuracy limits in normal
eyes. J Cataract Refract Surg.

[8] Aristodemou P, Knox Cartwright NE, Sparrow JM, Johnston RL. (2011). Formula choice: hoffer Q, Holladay 1, or SRK/T and refractive
outcomes in 8108 eyes after cataract surgery with biometry by partial
coherence interferometry. J Cataract Refract Surg.

[9] Haigis W. (2008). Intraocular lens calculation after refractive surgery for myopia:
Haigis-L formula. J Cataract Refract Surg.

[10] Joo J, Whang WJ, Oh TH, Kang KD, Kim HS, Moon JI. (2011). Accuracy of intraocular lens power calculation formulas in
primary angle closure glaucoma. Korean J Ophthalmol.

[11] Olsen T. (2007). Calculation of intraocular lens power: a review. Acta Ophthalmol Scand.

[12] Olsen T. (1992). Sources of error in intraocular lens power
calculation. J Ca taract Refract Surg.

[13] Murata C, Mallmann F, Yamazaki E, Campos M. (2007). Estudo do segmento anterior com a câmera rotatória
de Scheimpflug em pacientes candidatos à cirurgia
refrativa. Arq Bras Oftalmol.

[14] Mueller A, Thomas BC, Auffarth GU, Holzer MP. (2016). Comparison of a new image-guided system versus partial coherence
interferometry, Scheimpflug imaging, and optical low-coherence reflectometry
devices: keratometry and repeatability. J Cataract Refract Surg.

[15] Reitblat O, Levy A, Kleinmann G, Assia EI. (2018). Accuracy of intraocular lens power calculation using three
optical biometry measurement devices: the OA-2000, Lenstar-LS900 and
IOLMaster-500. Eye (Lond).

[16] Huang J, Pesudovs K, Wen D, Chen S, Wright T, Wang X (2011). Comparison of anterior segment measurements with rotating
Scheimpflug photography and partial coherence reflectometry. J Cataract Refract Surg.

[17] Németh G, Hassan Z, Módis L Jr, Szalai E, Katona K, Berta A (2011). Comparison of anterior chamber depth measurements conducted with
Pentacam HR^®^ and
IOLMaster^®^. Ophthalmic Surg Lasers Imaging.

[18] Utine CA, Altin F, Cakir H, Perente I. (2009). Comparison of anterior chamber depth measurements taken with the
Pentacam, Orbscan IIz and IOLMaster in myopic and emmetropic
eyes. Acta Ophthalmol.

[19] Zhao J, Chen Z, Zhou Z, Ding L, Zhou X. (2013). Evaluation of the repeatability of the Lenstar and comparison
with two other non-contact biometric devices in myopes. Clin Exp Optom.

[20] Visser N, Berendschot TT, Verbakel F, de Brabander J, Nuijts RM. (2012). Comparability and repeatability of corneal astigmatism
measurements using different measurement technologies. J Cataract Refract Surg.

[21] Eleftheriadis H. (2003). IOLMaster biometry: refractive results of 100 consecutive
cases. Br J Ophthalmol.

[22] Borasio E, Stevens J, Smith GT. (2006). Estimation of true corneal power after keratorefractive surgery
in eyes requiring cataract surgery: BESSt formula. J Cataract Refract Surg.

[23] Haigis W, Lege B, Miller N, Schneider B. (2000). Comparison of immersion ultrasound biometry and partial coherence
interferometry for intraocular lens calculation according to
Haigis. Graefes Arch Clin Exp Ophthalmol.

[24] Kawamorita T, Uozato H, Kamiya K, Bax L, Tsutsui K, Aizawa D (2009). Repeatability, reproducibility, and agreement characteristics of
rotating Scheimpflug photography and scanning-slit corneal topography for
corneal power measurement. J Cataract Refract Surg.

[25] Symes RJ, Say MJ, Ursell PG. (2010). Scheimpflug keratometry versus conventional automated keratometry
in routine cataract surgery. J Cataract Refract Surg.

